# Rivaroxaban for thromboprophylaxis after total hip or knee arthroplasty: a meta-analysis with trial sequential analysis of randomized controlled trials

**DOI:** 10.1038/srep23726

**Published:** 2016-03-29

**Authors:** Guang-Zhi Ning, Shun-Li Kan, Ling-Xiao Chen, Lei Shangguan, Shi-Qing Feng, Yue Zhou

**Affiliations:** 1 Department of Orthopaedics, Xinqiao Hospital, Third Military Medical University, Xinqiao Road, Shapingba District, Chongqing 400037, China; 2 Department of Orthopaedics, Tianjin Medical University General Hospital, 154 Anshan Road, Heping District, Tianjin 300052, China

## Abstract

Venous thromboembolism (VTE) is the most widespread severe complication after total hip arthroplasty (THA) and total knee arthroplasty (TKA). We conducted this meta-analysis to further validate the benefits and harms of rivaroxaban use for thromboprophylaxis after THA or TKA. We thoroughly searched PubMed, EMBASE, and the Cochrane Central Register of Controlled Trials. Trial sequential analysis (TSA) was applied to test the robustness of our findings and to obtain a more conservative estimation. Of 316 articles screened, nine studies were included. Compared with enoxaparin, rivaroxaban significantly reduced symptomatic VTE (P = 0.0001) and symptomatic deep vein thrombosis (DVT; P = 0.0001) but not symptomatic pulmonary embolism (P = 0.57). Furthermore, rivaroxaban was not associated with an increase in all-cause mortality, clinically relevant non-major bleeding and postoperative wound infection. However, the findings were accompanied by an increase in major bleeding (P = 0.02). The TSA demonstrated that the cumulative z-curve crossed the traditional boundary but not the trial sequential monitoring boundary and did not reach the required information size for major bleeding. Rivaroxaban was more beneficial than enoxaparin for preventing symptomatic DVT but increased the risk of major bleeding. According to the TSA results, more evidence is needed to verify the risk of major bleeding with rivaroxaban.

Venous thromboembolism (VTE), including deep vein thrombosis (DVT) and pulmonary embolism (PE), is the most widespread severe complication after total hip arthroplasty (THA) and total knee arthroplasty (TKA)^1^,^2^,^3^. Without thromboprophylaxis, the incidence of symptomatic DVT ranges from 15–30%, and the risk of symptomatic PE occurs in 0.5–2% of patients undergoing THA or TKA^4^,^5^,^6^,^7^. VTE results in the mortality of more than half a million people in Europe every year^8^.

Low molecular weight heparin (enoxaparin), synthetic pentasaccharides (fondaparinux), or vitamin K antagonists (warfarin) are recommended and used for routine postoperative thromboprophylaxis^9^,^10^,^11^. However, the need for daily subcutaneous injections, ongoing dose adjustments and laboratory monitoring have complicated their use^12^,^13^.

The drawbacks of existing anticoagulants have driven the development of new oral anticoagulants^14^: oral direct factor Xa inhibitors. Rivaroxaban is one of the first licensed novel oral direct factor Xa inhibitors. The National Institute of Clinical Excellence (NICE) and the American College of Chest Physicians (ACCP) have approved the use of rivaroxaban for thromboprophylaxis following THA and TKA^11^,^15^.

Several studies have assessed the benefits and harms of rivaroxaban for patients following THA or TKA. One study found that rivaroxaban was associated with a greater risk of major bleeding compared with enoxaparin^16^. Some systematic reviews have demonstrated that there was no difference in the incidence of major bleeding between rivaroxaban and enoxaparin and that rivaroxaban was more beneficial than enoxaparin for decreasing the risk of VTE. However, most of those reviews included only favorable rivaroxaban dosage^17^,^18^,^19^,^20^,^21^,^22^ or studies that reported phase III trials^23^,^24^. For these reasons, we conducted this systematic review and meta-analysis to further explicate the benefits and harms of rivaroxaban for thromboprophylaxis after THA or TKA. Furthermore, we applied trial sequential analysis (TSA) to test the robustness of our findings and to obtain a more conservative estimation.

## Results

### Study search

**Figure 1:**
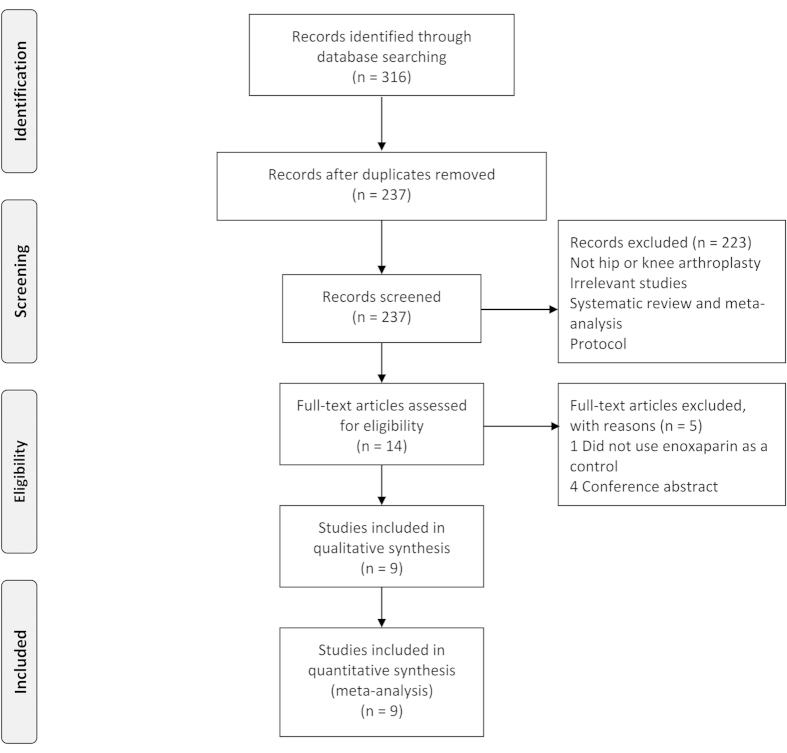
Flow diagram of study selection.

Figure 1 presents a summary of the study selection process. Of 316 articles screened, 301 were excluded because they were duplicates or did not meet the eligibility criteria. After verifying the full text of the remaining 15 articles, we discarded six studies. Finally, a total of nine studies met the eligibility criteria and were included in the quantitative analysis.

### Study characteristics

The study characteristics of the included studies are presented in Table 1. A total of nine randomized controlled trials^25^,^26^,^27^,^28^,^29^,^30^,^31^,^32^,^33^ were identified, of which seven^25^,^26^,^27^,^28^,^29^,^30^,^33^ used the enoxaparin regimen that is approved in Europe and two^31^,^32^ utilized the enoxaparin regimen approved in North America. Of the included studies, four used multiple dosages of rivaroxaban^25^,^26^,^27^,^32^; a single dosage of rivaroxaban was used in the remaining five trials^28^,^29^,^30^,^31^,^33^. The nine trials included 15,829 participants. Five^25^,^26^,^27^,^28^,^29^ of the trials compared rivaroxaban with enoxaparin for THA treatment; four trials^30^,^31^,^32^,^33^ compared the two treatments in TKA patients. Within these trials, 8781 participants were randomized to the rivaroxaban treatment, and 7048 patients were randomized to the enoxaparin treatment. All of the articles were reported in English between 2005 and 2014. The duration of follow-up ranged from 28 to 75 days. The mean age of the patients ranged between 62 and 68 years. There was a high proportion of females, ranging from 53–76%.

The major bleeding rates described in the four RECORD (Rosiglitazone Evaluated for Cardiac Outcomes and Regulation of Glycaemia in Diabetes) trials with rivaroxaban^28^,^29^,^30^,^31^ were 7–8 times lower than those of the enoxaparin groups in the remaining studies. The RECORD trials’ definition of major bleeding that excluded most bleeding from wounds, which differed from the definition of previous studies^34^,^35^, resulted in this phenomenon. This matter initially barred us from incorporating data on major bleeding into our report. However, an FDA review^36^ described the major bleeding rates of the RECORD trials without eliminating major wound bleedings. Therefore, the major bleeding data of the RECORD trials, as reported by the FDA, was utilized in this study.

### Risk of bias in the included studies

**Figure 2:**
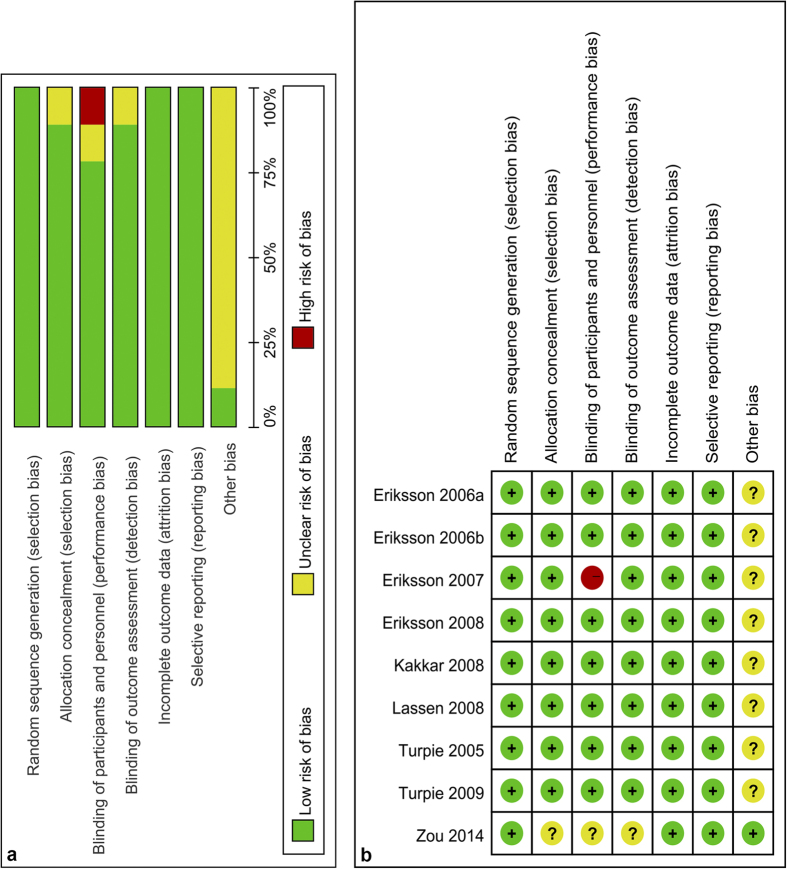
Risk of bias assessment of each included study. (**a**) Risk of bias graph. (**b**) Risk of bias summary.

One study^26^ was considered to be at high risk of bias. Eight studies^25^,^27^,^28^,^29^,^30^,^31^,^32^,^33^ had an unclear risk of bias. The high risk of bias was based on the use of the open-label method. Random sequence generation was carried out adequately in all the studies. Allocation concealment was adequate in eight studies^25^,^26^,^27^,^28^,^29^,^30^,^31^,^32^. Seven studies^25^,^27^,^28^,^29^,^30^,^31^,^32^ blinded the participants, personnel, and outcome assessors adequately. Seven trials^25^,^26^,^27^,^28^,^29^,^30^,^31^,^32^ were sponsored by a pharmaceutical company. The details about the risk of bias for each study are presented in Fig. 2.

### Quality of evidence assessment

Details regarding the Grading of Recommendations Assessment, Development and Evaluation (GRADE) evidence profiles for the outcomes are presented in Table 2. The GRADE level of evidence was low for symptomatic venous thromboembolism, major bleeding, and clinically relevant non-major bleeding; and moderate for all-cause mortality, symptomatic deep vein thrombosis, symptomatic pulmonary embolism, and postoperative wound infection.

## Primary outcomes

### Major bleeding

**Figure 3:**
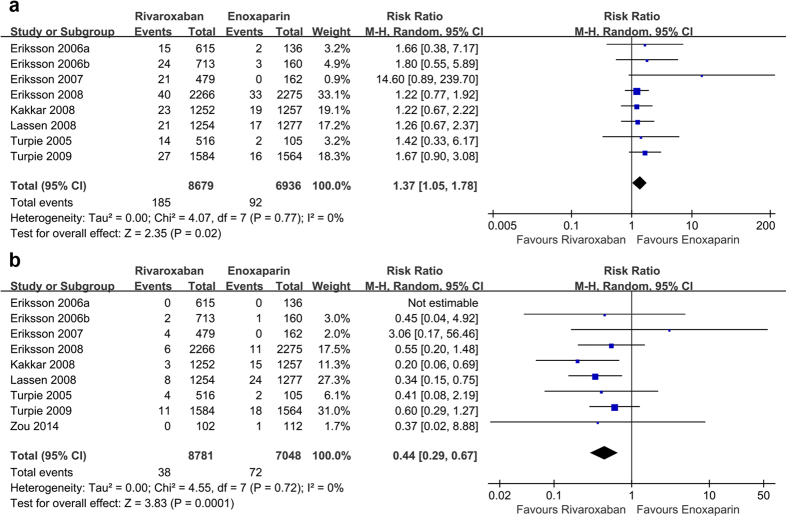
Forest plots of the included studies comparing major bleeding (a) and symptomatic venous thromboembolism (b) in patients who received rivaroxaban and those who received enoxaparin.

**Figure 4:**
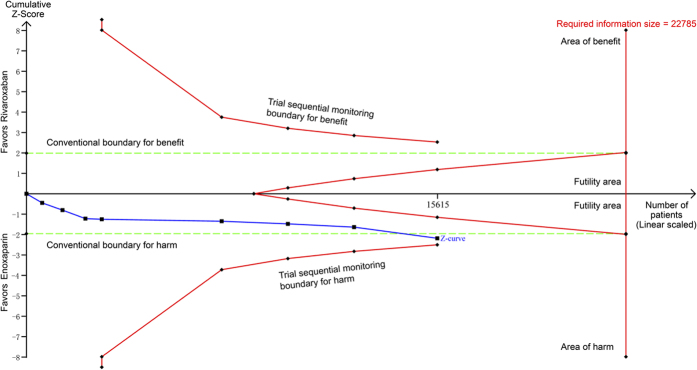
Trial sequential analysis of 8 trials comparing rivaroxaban with enoxaparin for major bleeding. Trial sequential analysis of 8 trials (black square fill icons) illustrating that The cumulative z-curve crossed the traditional boundary but not the trial sequential monitoring boundary and did not reach the required information size, suggesting the need for more evidence to establish additional harms of rivaroxaban over enoxaparin. A diversity adjusted required information size of 22,785 patients was calculated using α = 0.05 (two sided), β = 0.20 (power 80%), a relative risk reduction of −34.59% based on trials with adequate allocation concealment, and an event proportion of 1.33% in the control arm. X-axis: the number of patients randomized; Y-axis: the cumulative Z-Score; Horizontal green dotted lines: conventional boundaries (upper for benefit, Z-score = 1.96, lower for harm, Z-score = −1.96, two-sided P = 0.05); Sloping red full lines with black square fill icons: trial sequential monitoring boundaries calculated accordingly; Blue full line with black square fill icons: Z-curve; Vertical red full line: required information size calculated accordingly.

Eight randomized controlled trials^25^,^26^,^27^,^28^,^29^,^30^,^31^,^32^ randomized 15,615 participants and compared rivaroxaban with enoxaparin. Rivaroxaban was associated with a significant increase in the risk of major bleeding (relative risk (RR) = 1.37, 95% CI 1.05 to 1.78, P = 0.02; I^2^ = 0%; Fig. 3a) compared with enoxaparin. The cumulative z-curve crossed the traditional boundary but not the trial sequential monitoring boundary and did not reach the required information size, suggesting the need for more evidence to establish whether rivaroxaban is associated with greater harm compared with enoxaparin (Fig. 4).

### Symptomatic venous thromboembolism

**Figure 5:**
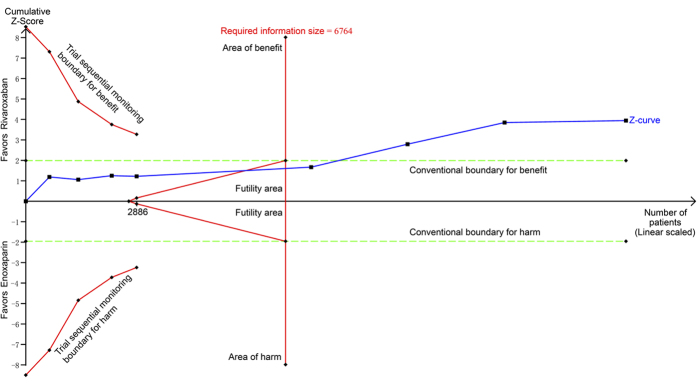
Trial sequential analysis of 9 trials comparing rivaroxaban with enoxaparin for symptomatic venous thromboembolism. Trial sequential analysis of 9 trials (black square fill icons) illustrating that the required information size had been reached and the cumulative z-curve crossed the traditional boundary, indicating further studies were not needed and were unlikely to change the inferences. A diversity adjusted required information size of 6,764 patients was calculated using α = 0.05 (two sided), β = 0.20 (power 80%), a relative risk reduction of 56.86% based on trials with adequate allocation concealment, and an event proportion of 1.02% in the control arm. X-axis: the number of patients randomized; Y-axis: the cumulative Z-Score; Horizontal green dotted lines: conventional boundaries (upper for benefit, Z-score = 1.96, lower for harm, Z-score = −1.96, two-sided P = 0.05); Sloping red full lines with black square fill icons: trial sequential monitoring boundaries calculated accordingly; Blue full line with black square fill icons: Z-curve; Vertical red full line: required information size calculated accordingly.

Nine studies^25^,^26^,^27^,^28^,^29^,^30^,^31^,^32^,^33^ including 15,829 participants reported data on symptomatic venous thromboembolism. Compared with enoxaparin, rivaroxaban significantly decreased symptomatic venous thromboembolism (RR = 0.44, 95% confidence interval (CI) 0.29 to 0.67, P = 0.0001; I^2^ = 0%; Fig. 3b). TSA demonstrated that the required information size had been reached and the cumulative z-curve crossed the traditional boundary, indicating further studies were not needed and would be unlikely to change the inferences (Fig. 5).

## Secondary Outcomes

**Figure 6:**
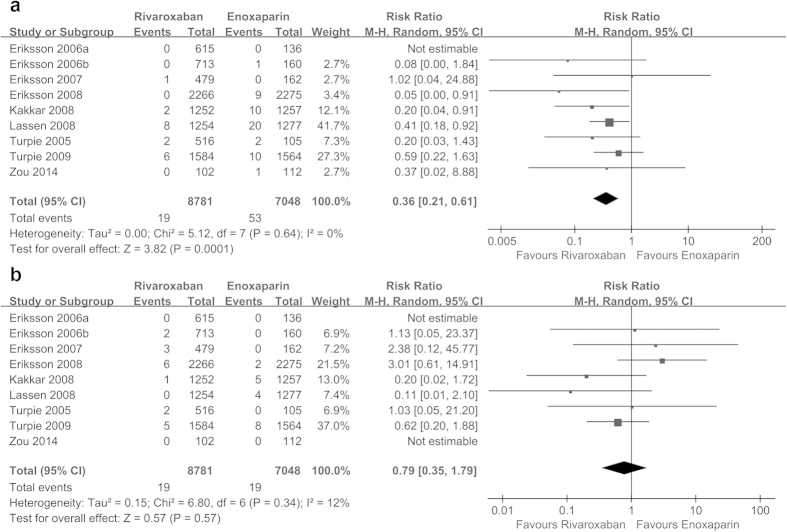
Forest plots of the included studies comparing symptomatic deep vein thrombosis (a) and symptomatic pulmonary embolism (b) in patients who received rivaroxaban and those who received enoxaparin.

Rivaroxaban reduced the incidence of symptomatic DVT (RR = 0.36, 95% CI 0.21 to 0.61, P = 0.0001; I^2^ = 0%; Fig. 6a) but not the risk of symptomatic PE (RR = 0.79, 95% CI 0.35 to 1.79, P = 0.57; I^2^ = 12%; Fig. 6b) compared with enoxaparin. Rivaroxaban was not different from enoxaparin in terms of the relative risk of all-cause mortality (RR = 0.63, 95% CI 0.27 to 1.44, P = 0.27), and there was no evidence of heterogeneity (see Supplementary Fig. S1). Similarly, neither rivaroxaban nor enoxaparin influenced the risk of clinically relevant non-major bleeding (RR = 1.23, 95% CI 1.00 to 1.51, P = 0.05; I^2^ = 0%; see Supplementary Fig. S2) and postoperative wound infection (RR = 0.97, 95% CI 0.57 to 1.66, P = 0.92; I^2^ = 0%; see Supplementary Fig. S3). The TSA for symptomatic DVT showed that the cumulative z-curve crossed the traditional boundary, and the required information size was reached, indicating that further studies were unlikely to change the inference (see Supplementary Fig. S4). The TSA for all-cause mortality and clinically relevant non-major bleeding showed that the cumulative z-curve did not cross the traditional boundary, suggesting that additional trials are needed to further verify the inferences (see Supplementary Figs S5 and S6). Because too little information could be used, no TSA could be conducted for symptomatic PE and postoperative wound infection.

### Subgroup analyses, sensitivity analyses, meta-regression analyses and publication bias

Subgroup analyses based on type of surgery (THA or TKA), allocation concealment (adequate or unclear), number of patients (<1000 or ≥1000), rivaroxaban dosage (a single dosage or multiple dosages), enoxaparin dosage (30 mg twice daily or 40 mg once daily), and surgery duration (<90 minutes or ≥90 minutes) did not exhibit noteworthy differences (see Supplementary Table S1). The decrease in the risk of symptomatic venous thromboembolism was higher in studies with adequate allocation concealment, ≥1000 patients, a single rivaroxaban dosage, an enoxaparin dosage of 40 mg once daily, and a surgery duration ≥90 minutes. The increase in the risk of major bleeding was higher in studies with adequate allocation concealment and a surgery duration ≥90 minutes.

On the whole, the sensitivity analyses did not change the findings; however, the exclusion of one study^31^ affected the result for major bleeding (see Supplementary Table S2).

Meta-regression analyses indicated that mean age was unlikely to be a source of heterogeneity in the results of this meta-analysis (see Supplementary Figs S7 and S8).

Because fewer than ten trials were included in our meta-analysis, we did not test the publication bias.

## Discussion

The present meta-analysis demonstrated that compared with enoxaparin, rivaroxaban significantly decreased the rate of symptomatic VTE in patients undergoing THA or TKA; additionally, it reduced the risk of symptomatic DVT but not symptomatic PE. The evidence of efficacy was verified by most subgroup analyses and the TSA. In addition, rivaroxaban was associated with a significant increase in major bleeding and a higher clinically relevant non-major bleeding tendency. There was no difference in all-cause mortality and postoperative wound infection.

This meta-analysis showed that rivaroxaban significantly increased the incidence of major bleeding, but not clinically relevant non-major bleeding, for patients with THA and TKA. Opina and colleagues^16^ concluded that rivaroxaban was associated with a 1.99-times higher RR for major bleeding compared with enoxaparin, which was consistent with the findings of this analysis. However, Gómez-Outes *et al*.^17^ and Cao *et al*.^22^ observed that the incidence of major bleeding did not significantly increase with rivaroxaban compared with enoxaparin. The major bleeding results reported in the four phase II clinical trials suggest that major bleeding mainly occurred in the high-dose rivaroxaban groups. Hence, the reason for the differing results may be that some reviews only considered the 10 mg once daily dosage of rivaroxaban in the phase II clinical trials^25^,^26^,^27^,^32^. Several studies have indicated that rivaroxaban was not associated with a significant increase in clinically relevant non-major bleeding^17^,^18^,^20^,^22^.

Rivaroxaban appeared more efficient than enoxaparin in preventing symptomatic VTE and symptomatic DVT; however, there was no significant difference in the risk of symptomatic PE. This finding was consistent among numerous studies^17^,^18^,^23^,^37^,^38^,^39^. The phase IV non-interventional study of rivaroxaban further verified that rivaroxaban significantly reduced the rate of symptomatic VTE^40^. Because of the low incidence of symptomatic PE following THA and TKA, additional large trials may be needed to reveal any underlying advantage of rivaroxaban for this result. In a previous meta-analysis, Gómez-Outes and colleagues found that rivaroxaban was associated with a significant decrease in symptomatic VTE and symptomatic DVT, but not symptomatic PE. Although these results were consistent with ours, this study only selected the 10 mg once daily dosage of rivaroxaban in four phase II clinical trials^25^,^26^,^27^,^32^, suggesting the possibility that the studies selected favorable results and presenting a possible loss of information^41^. The current analysis included all of the rivaroxaban dosages used in the phase II clinical trials; consequently, our findings are more reliable than those of analyses that excluded some dosages. Moreover, we included another new clinical trial^33^, which increased the statistical power. Furthermore, TSA was used in this meta-analysis to generate more conservative estimates. The TSA indicated that the present research provides ample and convincing evidence.

It has been confirmed that excessive anticoagulation may result in increased wound complications and postoperative infections^42^. However, our analysis did not support this hypothesis. Our study observed that there was no significant difference between rivaroxaban and enoxaparin in terms of postoperative wound infection and all-cause mortality. Furthermore, previous analyses have confirmed our findings^17^,^38^,^43^.

There are some highlights in this meta-analysis. The methodology recommended by the Cochrane Collaboration^41^ was applied. A thorough literature search of PubMed, EMBASE, and the Cochrane Central Register of Controlled Trials (CENTRAL) was performed without language restriction. Furthermore, two investigators independently appraised the risk of bias of the individual studies and assessed the quality of evidence using the GRADE approach for helping clinicians make clinical decisions. In addition, we utilized TSA to test the robustness of our findings and obtain a more conservative estimation.

Our study has several limitations. First, the trials were not consistent in terms of the duration of intervention. A short duration of treatment may affect the benefits and harms of rivaroxaban. Second, only two trials investigated the enoxaparin regimen of 30 mg daily. The inadequate number of patients in these two trials could not demonstrate a significant difference. Additionally, it was challenging to incorporate numerous trials within the same drug development program. However, because of the large number of participants and the fact that we included only randomized controlled trials, we believe that this analysis is convincing. Furthermore, because of the limited number of eligible studies, we did not assess publication bias. Our results should be interpreted with the consideration of the underlying publication bias. Finally, all of the studies were sponsored by pharmaceutical companies. This may also influence the robustness of our conclusions.

In conclusion, rivaroxaban was more beneficial than enoxaparin for preventing symptomatic VTE and symptomatic DVT, but not symptomatic PE, after THA and TKA. Moreover, rivaroxaban was not associated with an increase in all-cause mortality, clinically relevant non-major bleeding or postoperative wound infection. However, rivaroxaban was associated with an increase in major bleeding. According to the TSA results, more evidence is needed to verify the incidence of major bleeding associated with rivaroxaban.

## Methods

### Search strategy and study selection

We performed a thorough literature search of PubMed, EMBASE, and CENTRAL to identify randomized controlled trials that compared rivaroxaban with enoxaparin for patients undergoing THA or TKA. The literature search was completed on September 19, 2015. We combined MeSh terms with text words in the electronic search. The search terms pertaining to THA and TKA were combined with terms related to both rivaroxaban and enoxaparin. The details of the search strategies are presented in Supplementary Table S3. Language and publication date were not restricted. We also manually searched the study lists of all pertinent studies for additional relevant trials. Based on the titles and abstracts, two reviewers selected potential eligible studies, and the full text of the articles was examined for eligibility. Any disagreement was resolved through consensus.

### Eligibility criteria

1. Participants: Only trials enrolling adult patients undergoing THA or TKA were included in this meta-analysis.
2. Interventions: The intervention in the experimental group was, the new oral anticoagulant, rivoraxaban.
3. Comparisons: The intervention in the control group was the approved treatment, enoxaparin 40 mg once daily (Europe) or 30 mg twice daily (North America).
4. Outcomes: Studies were qualified when at least one of the following outcomes were described: symptomatic venous thromboembolism, major bleeding, all-cause mortality, clinically relevant non-major bleeding, and postoperative wound infection.
5. Study design: Only randomized controlled trials were considered as qualified in the present study.

### Data extraction and outcome measures

For the included studies, two assessors independently carried out the data extraction. Study characteristics, including details regarding methodology, patients, experimental and control interventions, and outcomes were extracted. For the outcomes of interest, we extracted the number of events and the total sample sizes. If the data were not described in the text of the articles, we extracted data from the tables and diagrams, if available^41^. The authors were contacted for extra information if sufficient information was not available from the text.

The primary outcome measures of interest were major bleeding and symptomatic VTE (symptomatic DVT or symptomatic PE). The secondary outcome measures consisted of all-cause mortality, symptomatic deep vein thrombosis, symptomatic pulmonary embolism, clinically relevant non-major bleeding, and postoperative wound infection.

Major bleeding was defined as clinically overt bleeding associated with a drop in the hemoglobin level of at least 2 g/dL; clinically overt bleeding resulting in the transfusion of ≥2 units of blood; fatal bleeding, bleeding into a critical organ (e.g., retroperitoneal, intracranial, or intraocular bleeding); or bleeding warranting treatment cessation or requiring re-operation. Clinically relevant non-major bleeding was defined as skin hematoma >25 cm^2^, wound hematoma >100 cm^2^, a multiple site or extrasurgical site bleed, epistaxis >5 min, and macoscopic hematuria >24 h^36^.

For studies with numerous intervention groups, we incorporated all pertinent experimental intervention groups (different rivaroxaban dosage regimens) into a specific group and combined all pertinent control intervention groups (different enoxaparin dosage regimens) into a specific control group. For binary results, both the sample sizes and the number of people with events can be totaled across groups. This method is recommended by the Cochrane Collaboration^41^.

### Risk of bias assessment

We used the risk of bias tool to evaluate all of the included studies in accordance with the Cochrane Handbook for Systematic Reviews of Interventions (version 5.1.0)^41^. Two investigators individualistically assessed all of the studies. The assessed domains included random sequence generation, allocation concealment, blinding of participants and personnel, blinding of the result assessor, incomplete result data, selective result reporting and other bias (baseline balance and fund). All of the domains were ranked as low risk of bias, high risk of bias, or unclear risk of bias. A trial was considered to have a high risk of bias if one or more key domains were considered to be at high risk. A trial was considered to have a low risk of bias if all key domains were considered to be at low risk. Otherwise, the studies were regarded as having an unclear risk of bias.

### Quality of evidence assessment

The quality of evidence for each pooled outcome was rated according to the GRADE system^44^. During the assessment process, the evidence for each pooled analysis was rated according to five major criteria: risk of bias, inconsistency, indirectness, imprecision and publication bias^44^,^45^. Each pooled analysis was defined as high, moderate, low, or very low quality. Two reviewers conducted the appraisals independently. Consensus was utilized to resolve any disagreement. GRADE Pro version 3.6 was used to generate summary tables.

### Statistical analysis

In this meta-analysis, RR and the corresponding 95% CI were calculated for all of the outcomes. The random-effect model was employed for the present meta-analysis^46^. The I^2^ statistic^47^ was used to assess the heterogeneity across studies. I^2^ > 50% was determined to indicate significant heterogeneity. Based on the type of surgery (THA or TKA), allocation concealment (adequate or unclear), number of patients (<1000 or ≥1000), rivaroxaban dosage (a single dosage or multiple dosages), enoxaparin dosage (30 mg twice daily or 40 mg once daily), and surgery duration (<90 minutes or ≥90 minutes), we conducted subgroup analyses for the primary outcomes. P < 0.05 indicated a statistically significant interaction between the estimates of the subgroups^48^. We performed the sensitivity analyses using a fixed-effect model and odds ratio (OR) with both random-effect and fixed-effect models, and by removing trials one by one. Furthermore, we carried out a meta-regression analysis to evaluate the potential effect of mean age on the primary outcomes. We used Egger’s linear regression test and funnel plots to test the publication bias when more than ten publications were included^49^. P values < 0.05 denoted statistically significant differences. We completed the statistical analyses using Review Manager version 5.3 (The Nordic Cochrane Centre, The Cochrane Collaboration, Copenhagen, 2014) and Stata version 12.0 (Stata Corp, College Station, TX).

### Trial Sequential Analysis

In a meta-analysis, the risk of false positive errors (type I error) may arise. This phenomenon may result from random errors when a small number of studies and participants is analyzed^50^,^51^,^52^ and repetitive statistical testing of the accumulation of additional data^52^,^53^. To correct for the incremental risk of type I errors, we used TSA to identify whether the findings of the cumulative meta-analysis were dependable and conclusive. TSA combines the required information size with trial sequential monitoring boundaries which adjust the confidence intervals and decrease type I errors^53^,^54^. When the cumulative z-curve crosses the trial sequential monitoring boundary or enters the futility area, an adequate level of evidence for the anticipated intervention effect may have been reached and no further trials are needed. If the z-curve does not cross any of the boundaries and the required information size has not been reached, the evidence is inadequate to reach a conclusion.

We estimated a diversity-adjusted information size in accordance with the diversity of the intervention effect estimates among the included studies. The TSA was conducted to maintain a type I error of 5% with a power of 80%. In the present meta-analysis, we calculated the required information size using the estimates of the intervention effects of trials with adequate allocation concealment^53^,^55^,^56^,^57^. Trial sequential analysis software version 0.9 beta (www.ctu.dk/tsa)^58^ was used for these analyses.

## Additional Information

**How to cite this article**: Ning, G.-Z. *et al*. Rivaroxaban for thromboprophylaxis after total hip or knee arthroplasty: a meta-analysis with trial sequential analysis of randomized controlled trials. *Sci. Rep*. **6**, 23726; doi: 10.1038/srep23726 (2016).

## Supplementary Material

Supplementary Information

## Figures and Tables

**Table 1:** Baseline characteristics of studies included in the meta-analysis.

Source	Intervention (dose, timing of first dose after surgery)		Type of surgery	Surgery duration (minutes)	Use of neuraxial anaesthesia (%)	No. of patients	Mean age (years), female (%), mean weight (kg)	Day of venography	Follow up (days)
	Experimental group	Control group							
Eriksson 2006a	Rivaroxaban 2.5, 5, 10, 20, or 30 mg twice daily, 5–9 days (6–8 hours)	Enoxaparin 40 mg once daily, 5–9 days (about 12 hours*)	THA	82	70	722	65, 59, 77	5–9	38–68
Eriksson 2006b	Rivaroxaban 10, 20, or 30 mg once daily, 5–9 days (6–8 hours)	Enoxaparin 40 mg once daily, 5–9 days (about 12 hours*)	THA	84	62	873	66, 64, 75	6–10	35–69
Eriksson 2007	Rivaroxaban 2.5, 5, 10, 20, or 30 mg twice daily, rivaroxaban 30 mg once daily, 5–9 days (6–8 hours)	Enoxaparin 40 mg once daily, 5–9 days (about 12 hours*)	THA	NA	73	641	64, 54, 79	5–9	38–68
Eriksson 2008	Rivaroxaban 10 mg once daily, 35d (6–8 hours)	Enoxaparin 40 mg once daily, 35 days (about 12 hours*)	THA	91	70	4541	63, 56, 78	36	66–71
Kakkar 2008	Rivaroxaban 10 mg once daily, 31–39 days (6–8 hours)	Enoxaparin 40 mg once daily, 14 days (about 12 hours*) +placebo 30 days	THA	93	71	2509	62, 53, 75	32–40	62–75
Lassen 2008	Rivaroxaban 10 mg once daily, 10–14 days (6–8 hours)	Enoxaparin 40 mg once daily, 10–14 days (about 12 hours*)	TKA	97	79	2531	68, 67, 81	11–15	41–50
Turpie 2005	Rivaroxaban 2.5, 5, 10, 20, or 30 mg twice daily, 5–9 days (6–8 hours)	Enoxaparin 30 mg twice daily, 5–9days (12–24 hours)	TKA	91	53	621	66, 55, 89	5–9	37–67
Turpie 2009	Rivaroxaban 10 mg once daily, 10–14 days (6–8 hours)	Enoxaparin 30 mg twice daily, 10–14 days (12–24 hours)	TKA	100	81	3148	65, 64, 84	11–15	40–49
Zou 2014	Rivaroxaban 10 mg once daily, 10–14 days (12 hours)	Enoxaparin 40 mg once daily, 14 days (12 hours)	TKA	85	100%	214	65, 76, NA	8–14†, 22–28†	28

THA: total hip arthroplasty; TKA: total knee arthroplasty; NA: not available.

*Administered preoperatively; other first doses were administered postoperatively.

^†^Color Doppler ultrasonography was performed.

**Table 2:** GRADE evidence profile.

Quality assessment							No of patients		Effect		Quality*	Importance
No of studies	Design	Risk of bias	Inconsistency	Indirectness	Imprecision	Other considerations	Rivaroxaban	Enoxaparin	Relative (95% CI)	Absolute		
Symptomatic venous thromboembolism (follow-up 28–75 days)												
9	randomised trials	serious^1^	no serious inconsistency	no serious indirectness	serious^2^	none	38/8781 (0.4%)	72/7048 (1%)	RR 0.44 (0.29 to 0.67)	6 fewer per 1000 (from 3 fewer to 7 fewer)	⊕⊕ΟΟ LOW	CRITICAL
								0.9%		5 fewer per 1000 (from 3 fewer to 6 fewer)		
Major bleeding (follow-up 35–75 days)												
8	randomised trials	serious^1^	no serious inconsistency	no serious indirectness	serious^3^	none	185/8679 (2.1%)	92/6936 (1.3%)	RR 1.37 (1.05 to 1.78)	5 more per 1000 (from 1 more to 10 more)	⊕⊕ΟΟ LOW	CRITICAL
								1.5%		6 more per 1000 (from 1 more to 12 more)		
All-cause mortality (follow-up 35–75 days)												
8	randomised trials	serious^1^	no serious inconsistency	no serious indirectness	no serious imprecision	none	10/8679 (0.1%)	15/6936 (0.2%)	RR 0.63 (0.27 to 1.44)	1 fewer per 1000 (from 2 fewer to 1 more)	⊕⊕⊕Ο MODERATE	CRITICAL
								0.1%		0 fewer per 1000 (from 1 fewer to 0 more)		
Symptomatic deep vein thrombosis (follow-up 28–75 days)												
9	randomised trials	serious^1^	no serious inconsistency	no serious indirectness	no serious imprecision	none	19/8781 (0.2%)	53/7048 (0.8%)	RR 0.36 (0.21 to 0.61)	5 fewer per 1000 (from 3 fewer to 6 fewer)	⊕⊕⊕Ο MODERATE	CRITICAL
								0.6%		4 fewer per 1000 (from 2 fewer to 5 fewer)		
Symptomatic pulmonary embolism (follow-up 28–75 days)												
9	randomised trials	serious^1^	no serious inconsistency	no serious indirectness	no serious imprecision	none	19/8781 (0.2%)	19/7048 (0.3%)	RR 0.79 (0.35 to 1.79)	1 fewer per 1000 (from 2 fewer to 2 more)	⊕⊕⊕Ο MODERATE	CRITICAL
								0%		—		
Clinically relevant non-major bleeding (follow-up 35–75 days)												
8	randomised trials	serious^1^	no serious inconsistency	no serious indirectness	serious^4^	none	251/8679 (2.9%)	156/6936 (2.2%)	RR 1.23 (1 to 1.51)	5 more per 1000 (from 0 more to 11 more)	⊕⊕ΟΟ LOW	IMPORTANT
								2.3%		5 more per 1000 (from 0 more to 12 more)		
Postoperative wound infection (follow-up 40–75 days)												
4	randomised trials	serious^5^	no serious inconsistency	no serious indirectness	no serious imprecision	none	27/6356 (0.4%)	28/6373 (0.4%)	RR 0.97 (0.57 to 1.66)	0 fewer per 1000 (from 2 fewer to 3 more)	⊕⊕⊕Ο MODERATE	CRITICAL
								0.4%		0 fewer per 1000 (from 2 fewer to 3 more)		

RR: relative risk.

^1^All the trials were judged to be at high risk of bias or unclear risk of bias.

^2^RR with 95% CI for one trial was 3.06 (0.17–56.46).

^3^RR with 95% CI for one trial was 14.60 (0.89–239.70).

^4^RR with 95% CI for one trial was 9.12 (0.55–149.84).

^5^All the trials were judged to be at unclear risk of bias.

^*^GRADE Working Group grades of evidence: high quality = further research is very unlikely to change our confidence in the estimate of effect; moderate quality = further research is likely to have an important impact on our confidence in the estimate of effect and may change the estimate; low quality = further research is very likely to have an important impact on our confidence in the estimate of effect and is likely to change the estimate; very low quality = we are very uncertain about the estimate.
