# Supplementary material for: Rivaroxaban for thromboprophylaxis after total hip or knee arthroplasty: a meta-analysis with trial sequential analysis of randomized controlled trials
Source: Sci Rep. 2016 Mar 29;6:23726. doi: 10.1038/srep23726 (PMC4810418; doi:10.1038/srep23726)
Supplement: Supplementary Information [file srep23726-s1.doc]

Rivaroxaban for thromboprophylaxis after total hip or knee arthroplasty: a meta-analysis with trial sequential analysis of randomized controlled trials

Guang-Zhi Ning^1,2^, Shun-Li Kan^2^, Ling-Xiao Chen^2^, Lei Shangguan^1^, Shi-Qing Feng^2^ & Yue Zhou^1^

^1^Department of Orthopaedics, Xinqiao Hospital, Third Military Medical University, Xinqiao Road, Shapingba District, Chongqing 400037, China;

^2^Department of Orthopaedics, Tianjin Medical University General Hospital, 154 Anshan Road, Heping District, Tianjin 300052, China

Correspondence and requests for materials should be addressed to Y.Z. (email: zhouyue2015xinqiao@163.com) or S.Q.F. (email: sqfeng@tmu.edu.cn)

Supplementary Table S1. Subgroup analyses of rivaroxaban compared with enoxaparin for symptomatic venous thromboembolism and major bleeding.

| Subgroup | Symptomatic Venous Thromboembolism | | | | Major Bleeding | | | |
| --- | --- | --- | --- | --- | --- | --- | --- | --- |
|  | No. Trials | RR (95% CI) | P Value | Test of Interaction, P | No. Trials | RR (95% CI) | P Value | Test of Interaction, P |
| Total | 9 | 0.44 (0.29-0.67) | 0.0001 | Not applicable | 8 | 1.37 (1.05-1.78) | 0.02 | Not applicable |
| Type of surgery |  |  |  |  |  |  |  |  |
| THA | 5 | 0.43 (0.19-0.96) | 0.04 | 0.92 | 5 | 1.32 (0.94-1.85) | 0.10 | 0.73 |
| TKA | 4 | 0.45 (0.27-0.76) | 0.002 |  | 3 | 1.45 (0.95-2.22) | 0.08 |  |
| Allocation concealment |  |  |  |  |  |  |  |  |
| Adequate | 8 | 0.45 (0.29-0.68) | 0.0002 | 0.90 | 5 | 1.31 (1.00-1.73) | 0.05 | 0.31 |
| Unclear | 1 | 0.37 (0.02-8.88) | 0.54 |  | 3 | 2.23 (0.83-6.03) | 0.11 |  |
| Number of patients |  |  |  |  |  |  |  |  |
| <1000 | 5 | 0.56 (0.18-1.80) | 0.33 | 0.66 | 4 | 1.92 (0.91-4.08) | 0.09 | 0.35 |
| ≥1000 | 4 | 0.43 (0.27-0.67) | 0.0002 |  | 4 | 1.31 (0.99-1.73) | 0.06 |  |
| Rivaroxaban dosage |  |  |  |  |  |  |  |  |
| Onefold dosage | 5 | 0.43 (0.27-0.66) | 0.0002 | 0.61 | 4 | 1.31 (0.99-1.73) | 0.06 | 0.35 |
| Multiple dosage | 4 | 0.60 (0.17-2.10) | 0.43 |  | 4 | 1.92 (0.91-4.08) | 0.09 |  |
| Enoxaparin dosage |  |  |  |  |  |  |  |  |
| 30 mg twice daily | 2 | 0.57 (0.29-1.12) | 0.10 | 0.38 | 2 | 1.63 (0.92-2.87) | 0.09 | 0.50 |
| 40 mg once daily | 7 | 0.38 (0.23-0.65) | 0.0004 |  | 6 | 1.31 (0.97-1.76) | 0.08 |  |
| Surgery duration |  |  |  |  |  |  |  |  |
| <90 minutes | 3 | 0.42 (0.06-2.83) | 0.37 | 0.98 | 2 | 1.74 (0.69-4.38) | 0.24 | 0.57 |
| ≥90 minutes | 5 | 0.43 (0.28-0.66) | 0.0001 |  | 5 | 1.31 (1.00-1.73) | 0.05 |  |

CI: confidence interval; RR: relative risk; THA: total hip arthroplasty; TKA: total knee arthroplasty.

**Supplementary Table S2. Sensitivity analyses.**

| Sensitivity analysis | Heterogeneity | | Effect | |
| --- | --- | --- | --- | --- |
|  | I^2^ | P | RR and 95%CI | P |
| **Major bleeding** |  |  |  |  |
| All studies (random-effect model with RR) | 0% | 0.77 | 1.37 (1.05, 1.78) | 0.02 |
| All studies (fixed-effect model with RR) | 0% | 0.77 | 1.45 (1.12, 1.89) | 0.005 |
| All studies (random-effect model with OR) | 0% | 0.76 | 1.38 (1.06, 1.80) | 0.02 |
| All studies (fixed-effect model with OR) | 0% | 0.76 | 1.46 (1.12, 1.90) | 0.005 |
| Exclude Eriksson 2006a | 0% | 0.68 | 1.36 (1.04, 1.78) | 0.02 |
| Exclude Eriksson 2006b | 0% | 0.70 | 1.35 (1.03, 1.77) | 0.03 |
| Exclude Eriksson 2007 | 0% | 0.98 | 1.34 (1.03, 1.75) | 0.03 |
| Exclude Eriksson 2008 | 0% | 0.71 | 1.45 (1.05, 2.01) | 0.02 |
| Exclude Kakkar 2008 | 0% | 0.69 | 1.41 (1.05, 1.89) | 0.02 |
| Exclude Lassen 2008 | 0% | 0.68 | 1.40 (1.05, 1.86) | 0.02 |
| Exclude Turpie 2005 | 0% | 0.67 | 1.37 (1.05, 1.79) | 0.02 |
| Exclude Turpie 2009 | 0% | 0.72 | 1.31 (0.98, 1.76) | 0.07 |
|  |  |  |  |  |
| **Symptomatic venous thromboembolism** |  |  |  |  |
| All studies (random-effect model with RR) | 0% | 0.72 | 0.44 (0.29, 0.67) | 0.0001 |
| All studies (fixed-effect model with RR) | 0% | 0.72 | 0.44 (0.29, 0.66) | <0.0001 |
| All studies (random-effect model with OR) | 0% | 0.71 | 0.44 (0.29, 0.67) | 0.0001 |
| All studies (fixed-effect model with OR) | 0% | 0.71 | 0.44 (0.29, 0.65) | <0.0001 |
| Exclude Eriksson 2006a | 0% | 0.72 | 0.44 (0.29, 0.67) | 0.0001 |
| Exclude Eriksson 2006b | 0% | 0.60 | 0.44 (0.29, 0.68) | 0.0002 |
| Exclude Eriksson 2007 | 0% | 0.83 | 0.43 (0.28, 0.65) | <0.0001 |
| Exclude Eriksson 2008 | 0% | 0.63 | 0.42 (0.27, 0.67) | 0.0002 |
| Exclude Kakkar 2008 | 0% | 0.84 | 0.49 (0.32, 0.76) | 0.002 |
| Exclude Lassen 2008 | 0% | 0.68 | 0.49 (0.30, 0.80) | 0.004 |
| Exclude Turpie 2005 | 0% | 0.60 | 0.45 (0.29, 0.69) | 0.0002 |
| Exclude Turpie 2009 | 0% | 0.73 | 0.39 (0.23, 0.64) | 0.0002 |
| Exclude Zou 2014 | 0% | 0.61 | 0.45 (0.29, 0.68) | 0.0002 |

CI: confidence interval; RR: relative risk; OR: odds ratio.

**Supplementary Table S3. Search strategies.**

**PubMed**

**Searched on:** September 19, 15

**Results: 78**

| **Search** | **Query** |
| --- | --- |
| #1 | "rivaroxaban" [Supplementary Concept] |
| #2 | ((rivaroxaban[Title/Abstract]) OR BAY 59-7939[Title/Abstract]) OR Xarelto[Title/Abstract] |
| #3 | "Arthroplasty, Replacement, Knee"[Mesh] |
| #4 | "Arthroplasty, Replacement, Hip"[Mesh] |
| #5 | ((arthroplast*[Title/Abstract]) OR replac*[Title/Abstract]) OR prosthe*[Title/Abstract] |
| #6 | "Knee Joint"[Mesh] OR "Knee"[Mesh] |
| #7 | (knee[Title/Abstract]) OR knee joint[Title/Abstract] |
| #8 | #6 OR #7 |
| #9 | "Hip"[Mesh] OR "Hip Joint"[Mesh] |
| #10 | (hip[Title/Abstract]) OR hip joint[Title/Abstract] |
| #11 | #9 OR #10 |
| #12 | #8 OR #11 |
| #13 | #5 AND #12 |
| 14 | #3 OR #4 OR #13 |
| 15 | #1 OR #2 |
| 16 | #14 AND #15 |
| 17 | "Randomized Controlled Trial" [Publication Type] OR "Randomized Controlled Trials as Topic"[Mesh] |
| 18 | random* |
| 19 | #17 OR #18 |
| 20 | #16 AND #19 |

**EMBASE**

**Searched on:** September 19, 15

**Results: 221**

| **Search** | **Query** |
| --- | --- |
| #1 | 'rivaroxaban'/exp |
| #2 | rivaroxaban:ab,ti |
| #3 | 'bay 59 7939':ab,ti |
| #4 | 'xarelto':ab,ti |
| #5 | #1 OR #2 OR #3 OR #4 |
| #6 | 'knee arthroplasty'/exp |
| #7 | 'hip arthroplasty'/exp |
| #8 | arthroplast*:ab,ti |
| #9 | replac* |
| #10 | prosthe*:ab,ti |
| #11 | #8 OR #9 OR #10 |
| #12 | 'knee'/exp |
| #13 | 'hip'/exp |
| 14 | 'knee':ab,ti |
| 15 | 'hip':ab,ti |
| 16 | #12 OR #13 OR #14 OR #15 |
| 17 | #11 AND #16 |
| 18 | #6 OR #7 OR #17 |
| 19 | #5 AND #18 |
| 20 | 'randomized controlled trial (topic)'/exp |
| 21 | 'randomized controlled trial'/exp |
| 22 | random* |
| 23 | #20 OR #21 OR #22 |
| 24 | #19 AND #23 |

**CENTRAL**

**Searched on:** September 19, 15

**Results: 17**

| **Search** | **Query** |
| --- | --- |
| #1 | rivaroxaban:ti,ab,kw or BAY 59-7939:ti,ab,kw or Xarelto:ti,ab,kw |
| #2 | MeSH descriptor: [Arthroplasty, Replacement, Knee] |
| #3 | MeSH descriptor: [Arthroplasty, Replacement, Hip] |
| #4 | arthroplast*:ti,ab,kw or replac*:ti,ab,kw or prosthe*:ti,ab,kw |
| #5 | MeSH descriptor: [Knee] |
| #6 | MeSH descriptor: [Knee Joint] |
| #7 | MeSH descriptor: [Hip] |
| #8 | MeSH descriptor: [Hip Joint] |
| #9 | #5 or #6 or #7 or #8 |
| #10 | #9 and #4 |
| #11 | #10 or #2 or #3 |
| #12 | #11 and #1 |
| #13 | MeSH descriptor: [Randomized Controlled Trial] |
| 14 | MeSH descriptor: [Randomized Controlled Trials as Topic] |
| 15 | random* |
| 16 | #13 or #14 or #15 |
| 17 | #12 AND #16 |


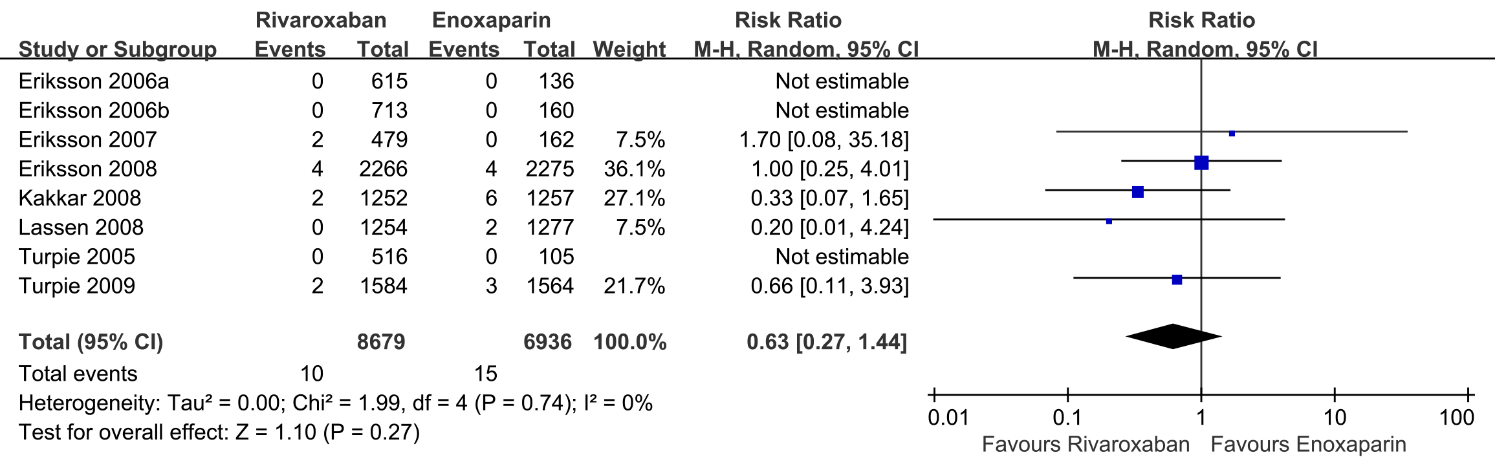


**Supplementary Figure. S1. Forest plots of the included studies comparing all-cause mortality in patients who received rivaroxaban and those who received enoxaparin.**


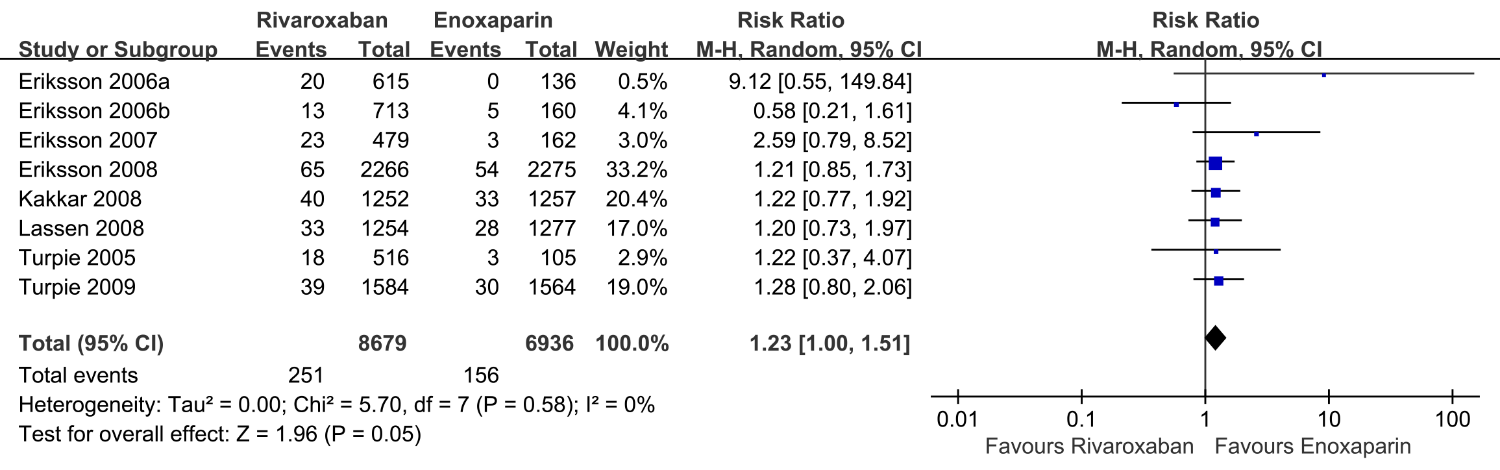


**Supplementary Figure. S2. Forest plots of the included studies comparing** **clinically relevant non-major bleeding in patients who received rivaroxaban and those who received enoxaparin.**


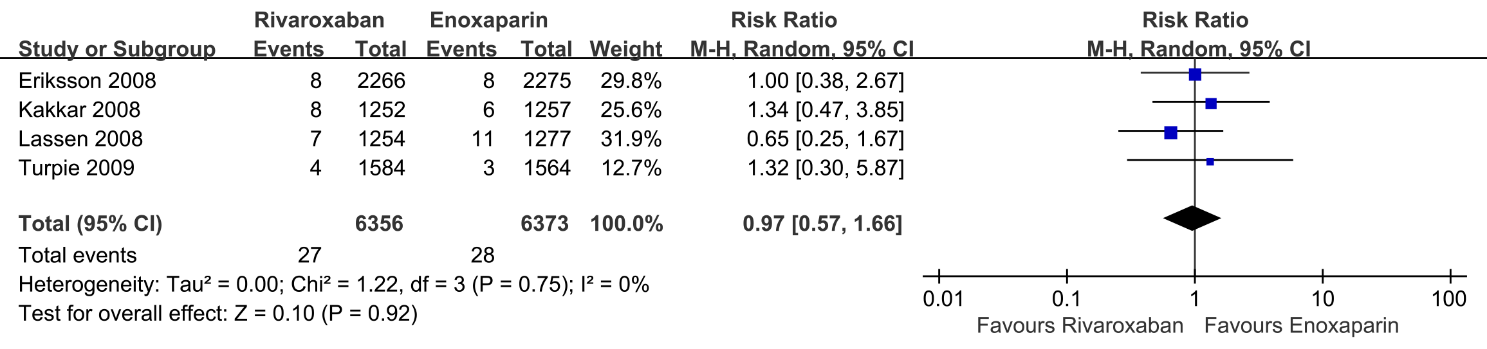


**Supplementary Figure. S3. Forest plots of the included studies comparing postoperative wound infection in patients who received rivaroxaban and those who received enoxaparin.**

**
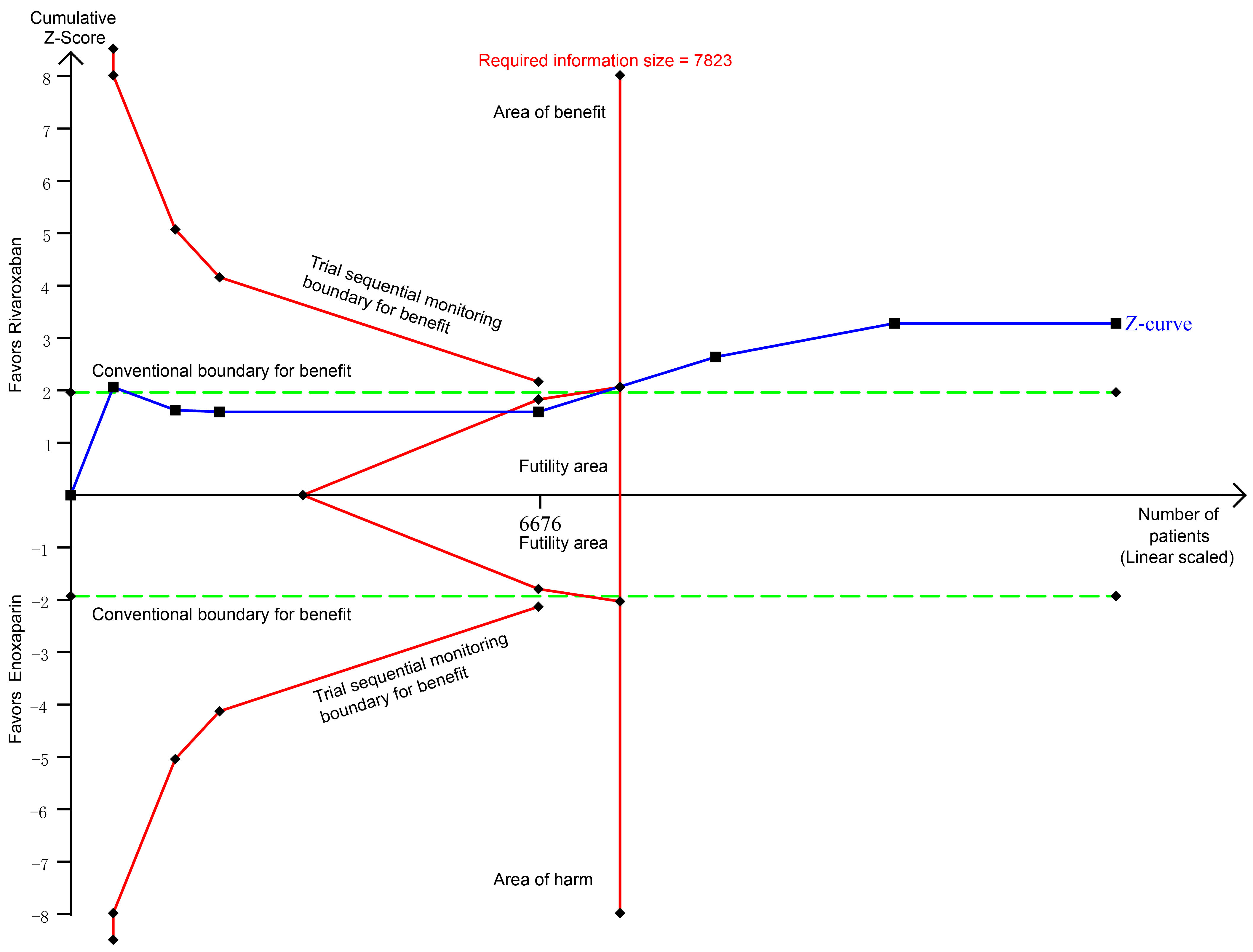
**

**Supplementary Figure. S4. Trial sequential analysis of 7 trials comparing rivaroxaban with enoxaparin for symptomatic deep vein thrombosis.** Trial sequential analysis of 7 trials (black square fill icons) illustrating that the cumulative z-curve crossed the traditional boundary and the required information size had been reached, indicating further studies were unlikely to change the inference. A diversity adjusted required information size of 7,823 patients was calculated using α = 0.05 (two sided), β = 0.20 (power 80%), a relative risk reduction of 60.53% based on trials with adequate allocation concealment, and an event proportion of 0.76% in the control arm. X-axis: the number of patients randomized; Y-axis: the cumulative Z-Score; Horizontal green dotted lines: conventional boundaries (upper for benefit, Z-score = 1.96, lower for harm, Z-score = -1.96, two-sided P = 0.05); Sloping red full lines with black square fill icons: trial sequential monitoring boundaries calculated accordingly; Blue full line with black square fill icons: Z-curve; Vertical red full line: required information size calculated accordingly.


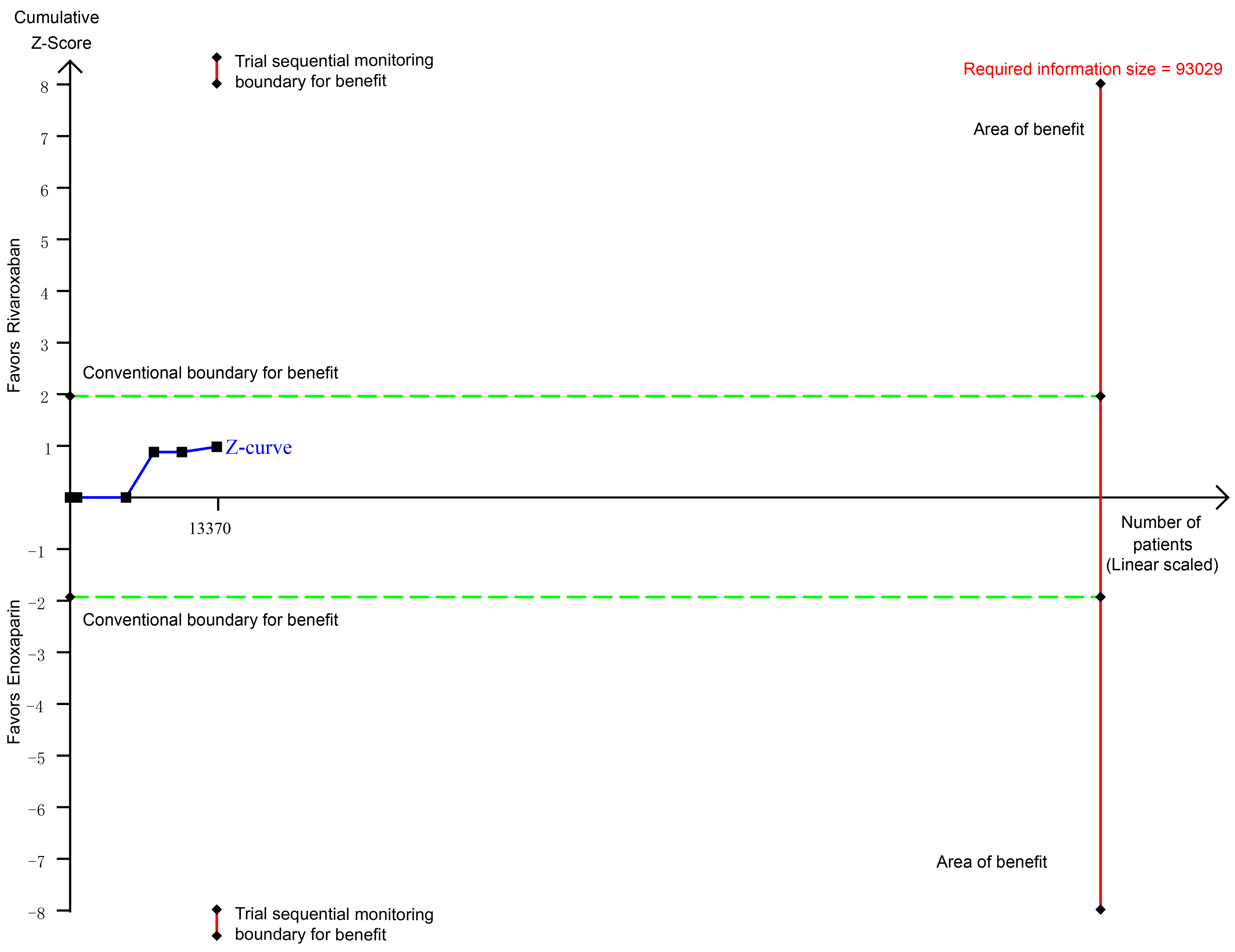


**Supplementary Figure. S5. Trial sequential analysis of 4 trials comparing rivaroxaban with enoxaparin for all-cause mortality.** Trial sequential analysis of 4 trials (black square fill icons) illustrating that the cumulative z-curve did not cross the traditional boundary, hinting that additional trials were needed to further verify the inferences. A diversity adjusted required information size of 93,029 patients was calculated using α = 0.05 (two sided), β = 0.20 (power 80%), a relative risk reduction of 34.78% based on trials with adequate allocation concealment, and an event proportion of 0.23% in the control arm. X-axis: the number of patients randomized; Y-axis: the cumulative Z-Score; Horizontal green dotted lines: conventional boundaries (upper for benefit, Z-score = 1.96, lower for harm, Z-score = -1.96, two-sided P = 0.05); Sloping red full lines with black square fill icons: trial sequential monitoring boundaries calculated accordingly; Blue full line with black square fill icons: Z-curve; Vertical red full line: required information size calculated accordingly.


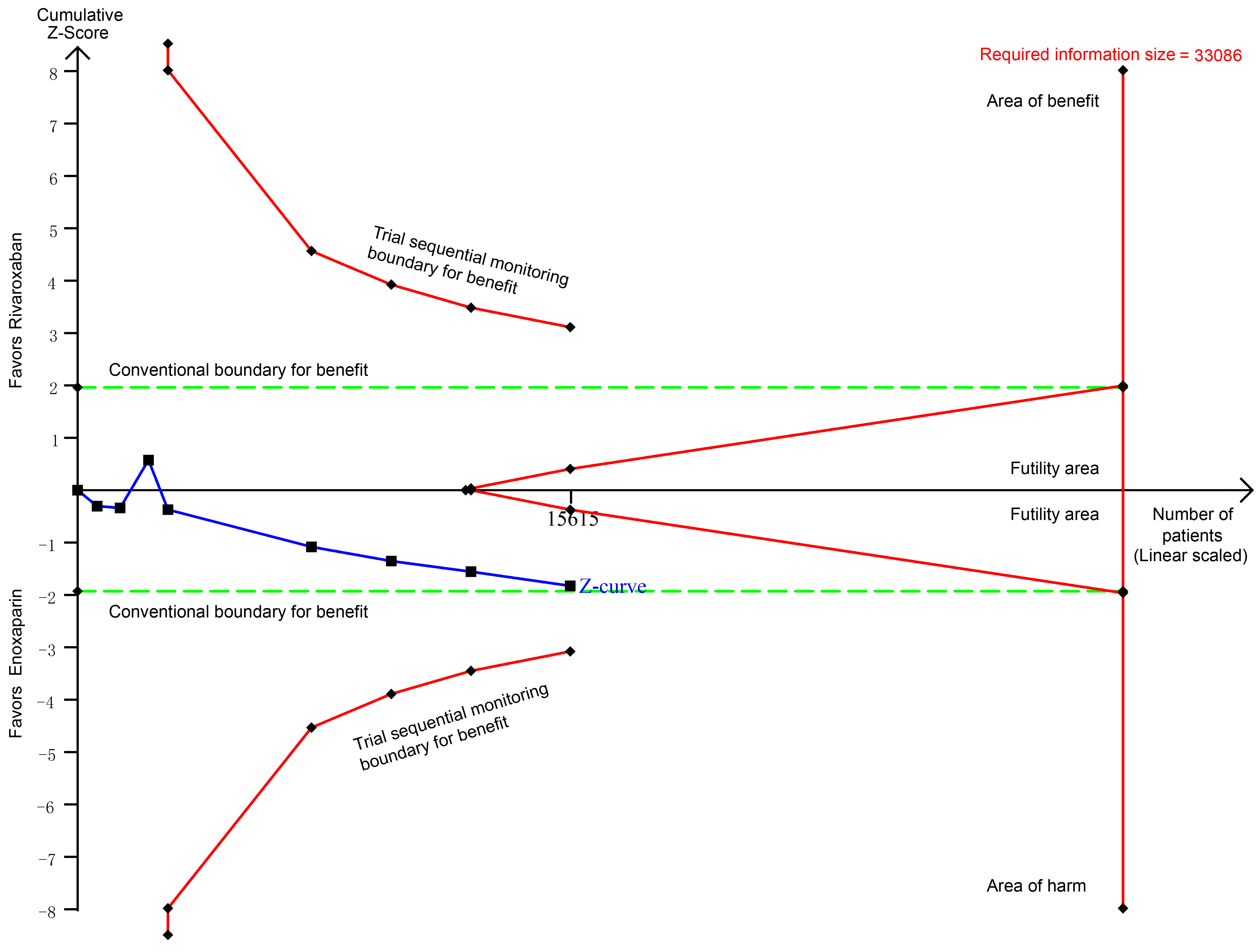


**Supplementary Figure. S6. Trial sequential analysis of 8 trials comparing rivaroxaban with enoxaparin for clinically relevant non-major bleeding.** Trial sequential analysis of 8 trials (black square fill icons) illustrating that the cumulative z-curve did not cross the traditional boundary, hinting that additional trials were needed to further verify the inferences. A diversity adjusted required information size of 33,086 patients was calculated using α = 0.05 (two sided), β = 0.20 (power 80%), a relative risk reduction of -21.33% based on trials with adequate allocation concealment, and an event proportion of 2.25% in the control arm. X-axis: the number of patients randomized; Y-axis: the cumulative Z-Score; Horizontal green dotted lines: conventional boundaries (upper for benefit, Z-score = 1.96, lower for harm, Z-score = -1.96, two-sided P = 0.05); Sloping red full lines with black square fill icons: trial sequential monitoring boundaries calculated accordingly; Blue full line with black square fill icons: Z-curve; Vertical red full line: required information size calculated accordingly.


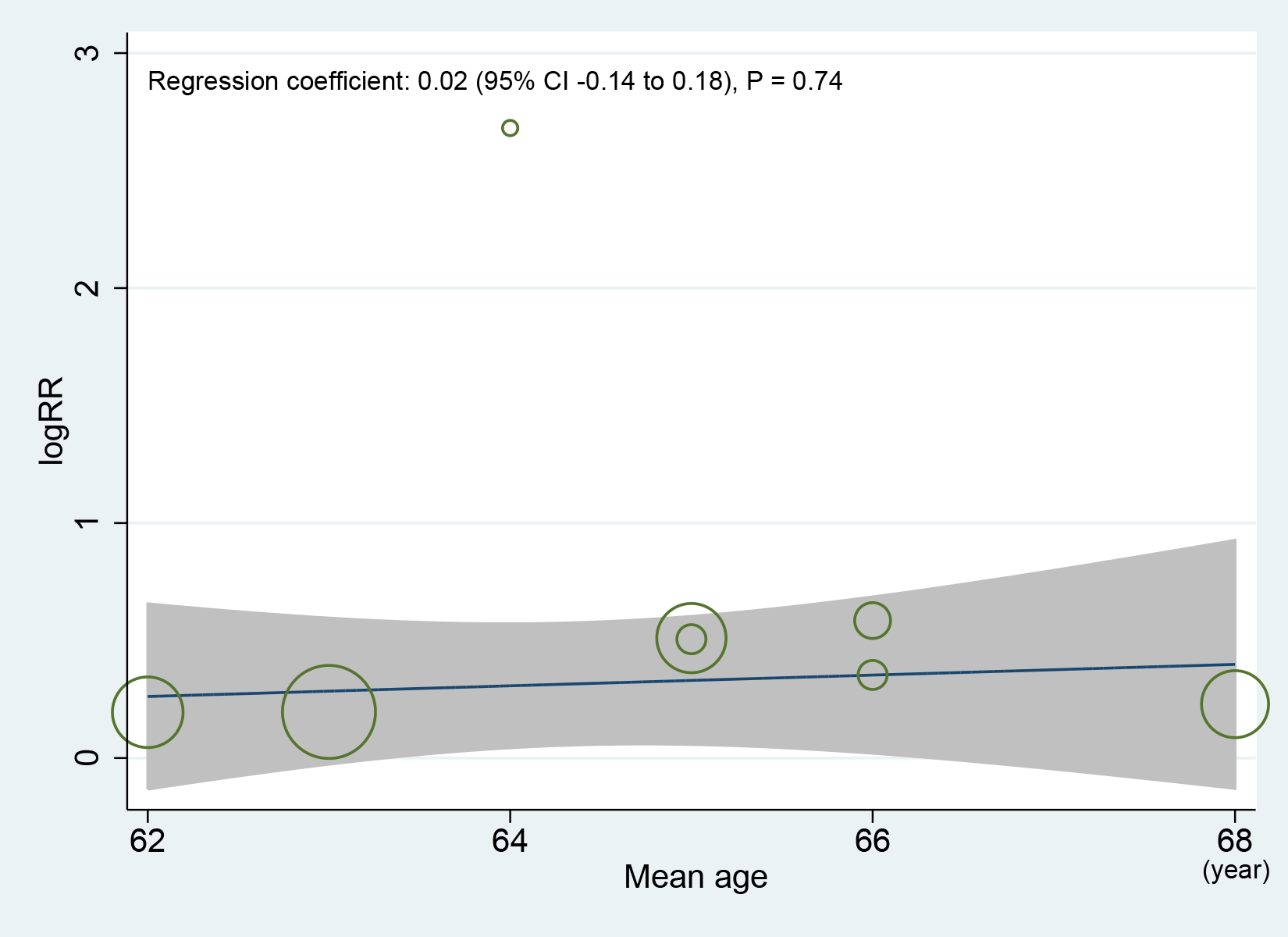


**Supplementary Figure. S7. Meta-regression analysis of influence of mean age on major bleeding rates of rivaroxaban treatment.** The circles represent each study. The size of the circle represents the power of the study. The solid line indicates the weighted regression line. RR: relative risk.


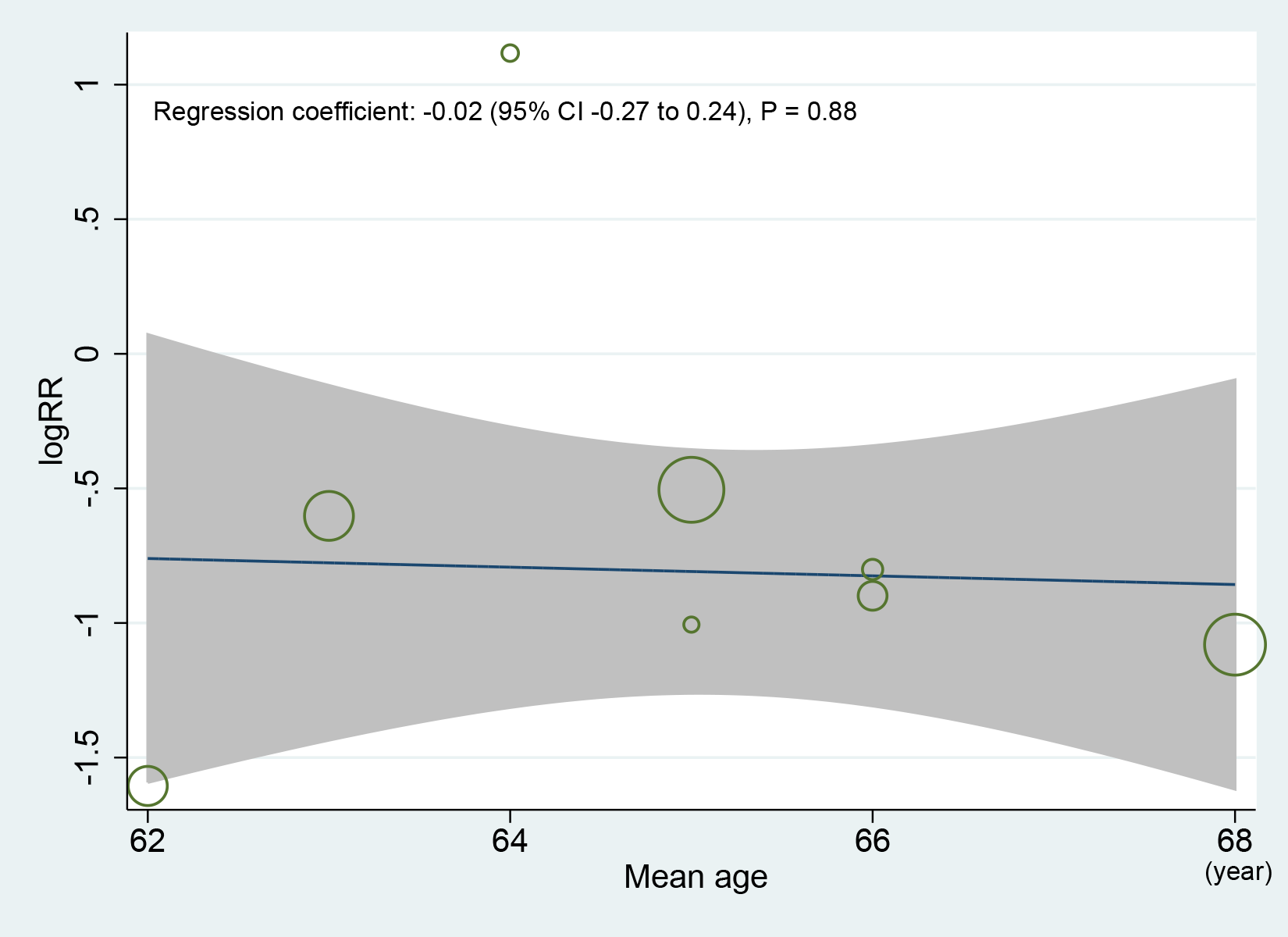


**Supplementary Figure. S8. Meta-regression analysis of influence of mean age on symptomatic venous thromboembolism rates of rivaroxaban treatment.** The circles represent each study. The size of the circle represents the power of the study. The solid line indicates the weighted regression line. RR: relative risk.
